# Absenteeism, Presenteeism, and the Economic Costs of Alcohol Hangover in The Netherlands

**DOI:** 10.3390/healthcare12030335

**Published:** 2024-01-29

**Authors:** Noortje R. Severeijns, Annabel S. M. Sips, Agnese Merlo, Gillian Bruce, Joris C. Verster

**Affiliations:** 1 Division of Pharmacology, Utrecht Institute for Pharmaceutical Sciences, Utrecht University, 3584 CG Utrecht, The Netherlands; n.r.severeijns@students.uu.nl (N.R.S.); annabelsophie.sips@gmail.com (A.S.M.S.); a.merlo@uu.nl (A.M.); 2 Division of Psychology and Social Work, School of Education and Social Sciences, University of the West of Scotland, Paisley PA1 2BE, UK; gillian.bruce@uws.ac.uk; 3 Centre for Human Psychopharmacology, Swinburne University, Melbourne, VIC 3122, Australia

**Keywords:** alcohol, hangover, absenteeism, presenteeism, work performance, economic costs

## Abstract

The alcohol hangover is defined as the combination of negative mental and physical symptoms that can be experienced after a single episode of alcohol consumption, starting when the blood alcohol concentration (BAC) approaches zero. Alcohol hangover symptoms such as fatigue, nausea, and headache can negatively affect daily activities, including work performance. The alcohol hangover can therefore be a cause of both absenteeism (not going to work) and presenteeism (going to work while hungover). An online survey among a convenience sample of *n* = 347 Dutch adults examined the number of days of absenteeism and presenteeism associated with having a hangover as well as the loss of productivity when going to work when hungover during the year 2019. In the Dutch sample, 8.1% of employees reported one or more days of absenteeism due to hangover in 2019, and 33.4% reported one or more days of presenteeism. The analyses revealed that alcohol hangover was associated with 0.2 days of absenteeism and 8.3 days of presenteeism and a productivity loss of 24.9% on days worked with a hangover. The estimated associated costs for the Dutch economy in 2019 of absenteeism (EUR 234,538,460) and presenteeism (EUR 2,423,603,184) total EUR 2,658,141,644. In conclusion, the alcohol hangover is associated with absenteeism, presenteeism, and reduced performance at work while hungover. As such, the annual costs of the alcohol hangover have a significant impact on the Dutch economy. However, these first findings on the economic costs of the alcohol hangover should be considered a rough estimate. They should be verified in a longitudinal study to minimize recall bias, including a nationally representative sample of sufficient sample size.

## 1. Introduction

In 2020, The Alcohol Hangover Research Group defined the alcohol hangover as the combination of negative mental and physical symptoms that can be experienced after a single episode of alcohol consumption, starting when the blood alcohol concentration (BAC) approaches zero [1]. Symptoms such as fatigue, nausea, and headache [2] may negatively impact cognitive and psychomotor functioning [3] and daily activities such as driving a car [4]. Alcohol hangover can be experienced by drinkers of both sexes and all ages [5,6]. At this time, no proven, effective, and safe hangover treatment is available [7]. Therefore, it is hypothesized that the hangover has a significant impact on daily activities, including being a cause of absenteeism (being absent from work due to having an alcohol hangover), presenteeism (being present at work while having a hangover), and performance at work.

McFarlin et al. [8] investigated absenteeism among 280 employed participants in the US and found that workers were roughly two times more likely to be absent from work the day after alcohol was consumed. A 2008 Australian survey [9] among 13,000 Australians found that 3.5% of all respondents reported missing at least one day of work per year due to alcohol use. Earlier studies found that presenteeism due to hangover was associated with poorer managerial skills [10], work-related problems such as conflicts with supervisors, lower productivity, and falling asleep [11], and an increased risk of work-related errors and accidents the day after alcohol consumption [11,12,13,14].

A UK study [15] investigated the economic costs of being hungover or intoxicated at work. Days of absenteeism and presenteeism were recorded, and the level of performance (compared to normal) when being hungover at work was measured. Of 3400 UK employees, 42% reported that they had gone to work hungover, with 9% stating that they had gone to work with a hangover at least once in the past six months. The overall annual costs associated with absenteeism and loss of performance (presenteeism) due to alcohol hangover and intoxication were estimated at 1.4 billion GBP. Unfortunately, the costs were not presented separately for hangover and intoxication. Thus far, no study has specifically investigated the economic costs of the alcohol hangover.

Studies unrelated to alcohol consumption have shown significant demographic differences in absenteeism and presenteeism. For example, compared with men, women usually report a higher number of absenteeism days [16,17,18] and presenteeism days [19,20,21]. In addition, Taimela et al. [22] found that young participants were more likely to be absent from work compared with older employees. Other research revealed that presenteeism rates are higher among younger, female workers. Possible explanations may be that women have a greater engagement with the workplace and a higher level of responsibility toward job performance than men [21,23]. With regard to educational background, it has been reported that individuals with a higher education status were less likely to report absenteeism [24] but more likely to report presenteeism [21J compared with workers with a lower educational background. Various explanations have been hypothesized for these differences, including different job types with different responsibilities [21], differences in job satisfaction [21], and the fact that less-educated individuals more often have an unhealthy lifestyle [24], including higher levels of alcohol consumption.

The current study aimed to evaluate the rate of absenteeism and presenteeism due to alcohol hangover in 2019. In addition, performance levels and average hangover severity were assessed for absenteeism and presenteeism days. To investigate these effects in more detail, analyses were also conducted to evaluate the impact of the demographic variables sex, age, career level, and education level. It was hypothesized that absenteeism rates would be higher among young, female, less-educated individuals and presenteeism rates would be higher among younger, female, more-educated individuals. A significant negative relationship was expected between the average hangover severity and the performance level reduction on presenteeism days.

## 2. Materials and Methods

Data were taken from an online survey conducted among the general Dutch population aged 18 years and older [25]. The survey was conducted between 24 June and 26 July 2020. There were no other inclusion or exclusion criteria. The survey was designed using SurveyMonkey and potential participants were invited to complete the survey through Facebook advertising. Ethical approval for the study was granted by the Ethics Committee of the Faculty of Social and Behavioural Sciences of Utrecht University (approval code: FETC17-061) and all participants gave electronic informed consent. The methodology and content of the survey are described elsewhere in detail [25].

For the current analyses, participants were only included if (a) they reported having had a job in 2019 and (b) they reported when they consumed alcohol in 2019. Sex and age were recorded, and participants indicated their highest level of education. A list of education types from Statistics Netherlands was used for this purpose [26]. Job type was recorded using the job categorization of Statistics Netherlands [27]. Career level was defined as junior (18–34 years old), middle (35–50 years old), and senior (51–65 years old). Absenteeism and presenteeism due to alcohol hangover were assessed for 2019. Questions were adapted from a study by the UK Institute of Alcohol Studies (IAS) that estimated the costs of workplace hangovers and intoxication for the UK economy [15]. Questions concerned the number of days in 2019 of (a) absenteeism, i.e., the number of days not worked due to having a hangover, and (b) presenteeism, i.e., the number of days worked while having a hangover. Regarding presenteeism, participants could further indicate, in comparison to a regular working day without having a hangover, how well they performed at work on days when they had an alcohol hangover. The performance level was rated on a scale ranging from 0% (‘compared to a regular day I achieved nothing/did not work’) to 100% (‘my work was absolutely not influenced by experiencing reduced immune fitness’). Finally, for both absenteeism days and presenteeism days, participants rated their average hangover severity on a scale ranging from 0 (absent) to 10 (extreme) [28].

### Statistical Analysis

A total of *n* = 1910 individuals completed the study. Of these, *n* = 365 consumed alcohol, were 18–65 years old, and had a job in the year 2019. These participants comprised the final sample used for the analysis.

Statistical analyses were conducted with SPSS (IBM Corp. Released 2013. IBM SPSS Statistics for Mac, Version 29.0. Armonk, NY, USA: IBM Corp.). Means and the standard deviation (SD) were calculated, and the distribution of the means was checked for normality. Since the data were not normally distributed, non-parametric tests were applied. Percentual differences were evaluated with chi-squared tests. Differences in means between groups were evaluated with the Independent-Samples Mann–Whitney U-test (2 groups, e.g., sex) or the Independent-Samples Kruskal–Wallis test, applying Bonferroni’s correction for multiple comparisons (more than 2 groups, e.g., education level).

**Figure 1:**
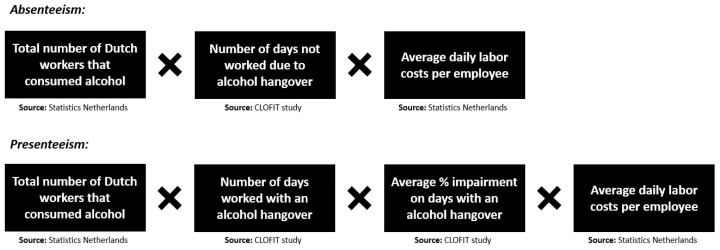
Calculation of the economic costs of alcohol hangover due to absenteeism and presenteeism. Data were collected for 2019 (the year prior to the COVID-19 pandemic). CLOFIT Study, ‘Corona: how fit are you?’ Study [25,32,33,34,35,36].

Differences in proportions between men and women were computed with the N-1 Chi-squared test as recommended by Campbell [29] and Richardson [30] (available at https://www.medcalc.org/calc/comparison_of_proportions.php, accessed 20 November 2023). Spearman’s correlations were computed between hangover severity and the number of days of absenteeism and presenteeism and the performance level on days worked with a hangover. Finally, the economic cost of alcohol hangover was estimated, applying the methodology used by Bhattacharya [15], which we successfully used in a previous study [31]. The formulas used to calculate the economic costs of absenteeism and presenteeism are summarized in Figure 1.

The number of employees in 2019 was obtained from Statistics Netherlands [32]. Of them, 8,886,000 individuals were between the ages of 15 and 65 years old. To estimate the number of employees between 18 and 65 years old, a correction was made (3/5 of the number of employees between 15 and 20 years old (692,000) was used to estimate the 18–20-year-old group. This yielded 8,886,000 − (3/6 × 692,000) = 8,540,000 employees between 18 and 65 years old. The percentage of the Dutch adult population that consumed alcohol in 2019 was estimated at 79.1% by Statistics Netherlands [33] Thus, the total number of Dutch employees that consumed alcohol was estimated at 0.791 × 8,540,000 = 6,755,140 employees.

Data from Statistics Netherlands revealed that the average income (before income tax) per employee in 2018 equaled EUR 44,000 [34]. Applying an inflation correction of 2.6% [35], the average yearly income for 2019 was estimated at EUR 45,144 (EUR 868.15 per week). On average, employees worked 31 h per week (6.2 h per day assuming 5 working days) [36]. The income per hour was calculated and equaled EUR 28. Together, the average daily labor costs were estimated at EUR 28 × 6.2 h = EUR 173.6 per day. The costs of alcohol hangover for the Dutch economy were estimated by calculating the sum of costs due to absenteeism and presenteeism.

## 3. Results

The dataset comprised *n* = 347 participants with a mean (SD) age of 35.5 (14.6) years old, and 63.4% were female. On average, participants worked 29 h per week divided over 4.2 working days. Their demographics are summarized in Table 1.

Females were significantly younger than males, more often worked at the junior career level, and worked fewer hours per week than males. No significant sex differences were found for education level, job category, or underlying disease status. In the sample, participants with a higher education level at the junior career level were over-represented.

Data on absenteeism, presenteeism, and work performance while hungover are summarized in Table 2. Of the sample, 8.1% of employees reported one or more days of absenteeism due to hangover in 2019, and 33.4% reported one or more days of presenteeism. The alcohol hangover was associated with 0.2 days of absenteeism and 8.3 days of presenteeism. The performance reduction while working with a hangover was estimated at 24.9%. The hangover severity on absenteeism days was significantly higher than the hangover severity on presenteeism days (*p* < 0.001). No significant sex differences were found.

Significant correlations were found between hangover severity and the number of days of absenteeism (r = 0.629, *p* < 0.001) and the number of days of presenteeism (r = 0.349, *p* < 0.001. The correlation between hangover severity and performance level on presenteeism days did not reach statistical significance (r = −0.173, *p* = 0.062).

The results according to career level are summarized in Table 3. Compared with individuals at the junior career level, individuals at the middle career level reported significantly more absenteeism days (*p* = 0.021) and individuals at the middle and senior levels reported significantly more days of presenteeism (all *p* < 0.001). However, the performance loss on hangover days was significantly greater (*p* < 0.001) for individuals at the junior career level (−27.2%) compared with individuals at the middle career level (−19.3%) and the senior career level (−20.0%).

Significant correlations were found between age and the number of absenteeism days (r = −0.132, *p* = 0.014) and between age and the number of presenteeism days (r = −0.309, *p* < 0.001). Age did not correlate significantly with the reported average hangover severity on these days.

Table 4 shows the results according to education level. Compared to the low education group, no significant differences were found for any of the assessed variables.

### Estimated Costs

**Figure 2:**
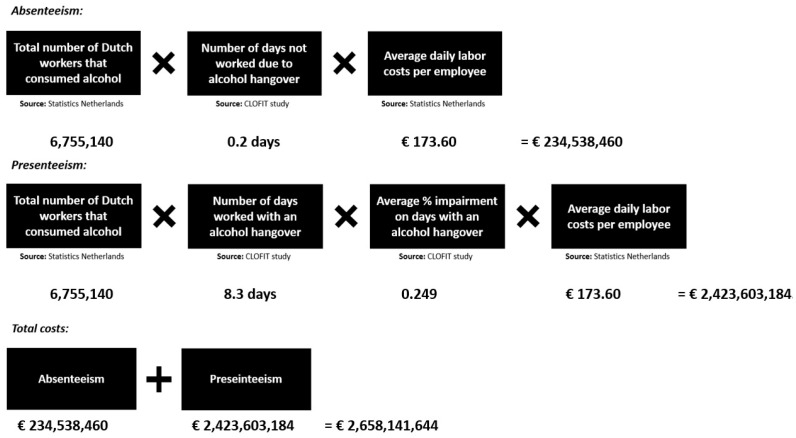
Estimated costs of alcohol hangover for the Dutch economy. Data were collected for 2019 (the year prior to the COVID-19 pandemic). CLOFIT Study, ‘Corona: how fit are you?’ Study [25,32,33,34,35,36].

The alcohol-hangover-related costs of absenteeism and presenteeism for the Dutch economy were estimated by applying the methodology used by Bhattacharya [15]. The calculations are summarized in Figure 2. The costs of absenteeism were estimated at EUR 234,538,460 and the costs of presenteeism at EUR 2,423,603,184. The overall costs of the alcohol hangover for the Dutch economy in 2019 were estimated at EUR 2,658,141,644.

## 4. Discussion

Previous research from the UK revealed that the alcohol hangover was associated with significant rates of absenteeism and presenteeism and a considerable loss of productivity at the workplace [15]. The current study among Dutch employees confirmed these findings. In the Dutch sample, 8.1% of employees reported one or more days of absenteeism due to hangover in 2019, and 33.4% reported one or more days of presenteeism. In 2019, 0.2 days of absenteeism and 8.3 days of presenteeism were related to alcohol hangover, and a productivity loss of 24.9% was reported for days worked with a hangover. The estimated associated costs for the Dutch economy in 2019 of absenteeism (EUR 234,538,460) and presenteeism (EUR 2,423,603,184) comprise EUR 2,658,141,644.

The substantial loss of productivity on presenteeism is understandable when considering the fact the alcohol hangover has been associated with cognitive impairments such as memory loss, impaired psychomotor functioning, and negative mood effects [37,38,39,40]. Other research reported poorer academic functioning during the hangover period [41]. It is of concern that these negative effects on productivity and work performance are not always recognized by hungover workers. For example, a study among professional truck drivers in the Netherlands revealed that 75.7% of them reported driving better while hungover than the average sober driver [42], while experimental studies showed that driving while hungover is significantly impaired [4].

There are no other studies that specifically investigated absenteeism and presenteeism due to the alcohol hangover. One study examined associated costs and presented the combined costs of alcohol hangover and intoxication [15] but did not specify the costs due to hangover alone. To put the estimated economic costs of alcohol hangover for the Dutch economy into perspective, these were more than three times higher than the Dutch government spent on infrastructure and water management (EUR 820,491,000) and agriculture, nature, and food quality (EUR 851,137,000) and approached the 2019 budget spent on foreign trade and development aid (EUR 2,994,876,000) [43]. In 2019, the Dutch budget for public health, welfare, and sports equaled EUR 3,936,745,000 [43]. The latter underlines the significant impact of the alcohol hangover on the Dutch economy.

In another study, we estimated the 2019 economic costs of reduced immune fitness [31]. Immune fitness refers to the capacity of the body to respond to health challenges (such as infections) by activating an appropriate immune response, which is essential to maintaining health, preventing and resolving disease, and improving quality of life [44]. An average of 2.9 absenteeism days and 19 presenteeism days were reported, with a performance reduction of 22.8% when working on days with reduced immune fitness. The associated costs of reduced immune fitness were estimated at EUR 10.7 billion (EUR 4.3 billion for absenteeism and EUR 6.4 billion for presenteeism). While the percentage of performance reduction corresponds to that found for the alcohol hangover, fewer absenteeism and presenteeism days were reported compared with those associated with reduced immune fitness. The latter is understandable, as reduced immune fitness can be the consequence of a variety of different diseases and health conditions.

In the current study, most absenteeism days were reported by individuals at the middle career level, whereas most presenteeism days were reported by individuals at the senior career level. However, the performance loss on presenteeism days was significantly greater for individuals at the junior career level (−27.2%) compared with individuals at the middle career level (−19.3%) and the senior career level (−20.0%). On first sight, these findings seem not to be in line with previous studies showing that younger (in particular male) individuals consume more alcohol than older individuals [45] and are thus more susceptible to experiencing hangovers [6]. Indeed, studies have shown a decline in the number of hangovers experienced when growing older [46,47]. However, it must be stressed that these studies often comprise the whole population (both employed and unemployed individuals), in which younger individuals without a job outnumber young individuals with a job. Research has shown differences in drinking behavior between students and non-students [48,49,50]. The transition to adulthood (e.g., career entry or starting a family) may have a moderating effect on alcohol consumption in order to adapt to changing daytime activities and new obligations [51].

The absence of significant sex differences is also noteworthy. Men usually consume more alcohol than women and more frequently experience hangovers, whereas women may be more sensitive to alcohol effects [5]. The literature yields inconsistent results as previous studies reported both lower and higher rates of absenteeism and presenteeism among women [21,52,53]. The use of a convenience sample and the type of occupation of participants may have an influence on study outcomes. The current sample size was too small to compare different occupation groups, and people working in hospitality and healthcare were over-represented.

This is the first study estimating the economic costs of the alcohol hangover. However, the study has several limitations and, therefore, the estimated amount must be considered only as a rough estimate. Firstly, the data were retrospectively self-reported and therefore subject to possible recall bias. Secondly, the relatively small convenience sample is not fully representative of the overall Dutch working population. For example, the distribution of job categories of the current sample was different from that of the general Dutch population, and participants with a higher education level and women were over-represented. Only a minority of the total study sample had a job in 2019 and could be included in the analysis. The fact that the sample was recruited via Facebook may have influenced the characteristics of the study sample, as not all age groups are equally represented on social media. Thirdly, besides absenteeism and presenteeism, no additional economic costs were considered. However, previous studies have shown that being hungover or drinking alcohol at work has negative consequences for co-workers [13,14,15,54]. Finally, the calculations used a simple model and, therefore, may be less accurate. For example, the average population labor costs and working hours were estimated for the sample instead of the actual costs per person. Applying other models to calculate hangover costs, including different additional variables (e.g., healthcare costs), may result in a different, presumably higher, cost estimate. Taken together, the fact that this study used a convenience sample likely had a significant influence on the study outcome. Therefore, future studies should verify the current findings using more precise calculations and a larger nationally representative sample. The use of a longitudinal study design, allowing for momentary assessments, also will enable us to more accurately assess absenteeism, presenteeism, and performance levels, as this minimizes recall bias. The use of a nationally representative sample with a sufficient sample size would also allow us to calculate the economic costs according to sex, age group, industry type, and education level.

## 5. Conclusions

Notwithstanding the limitations of the study, the implications of the alcohol hangover for the Dutch economy are evident. That is, the current study showed that the alcohol hangover is associated with absenteeism, presenteeism, and a significant reduction in work productivity, all of which are associated with significant costs for the Dutch economy. It is, therefore, important to proactively address the issue of alcohol hangover in the workplace context and to create awareness among the general public in order to reduce excessive alcohol consumption and subsequent hangovers.

## Figures and Tables

**Table 1:** Demographics.

Variables Assessed	Overall	Men	Women	*p*-Value
*n* (%)	347 (100%)	127 (36.6%)	220 (63.4%)	<0.001 *
Age (Mean, SD)	35.5 (14.6)	39.6 (15.2)	33.1 (13.7)	<0.001 *
Career level				
Junior (18–34 years old), *n* (%)	214 (61.7%)	62 (48.8%)	152 (69.1%)	0.005 *
Middle (35–50 years old), *n* (%)	58 (16.7%)	26 (20.5%)	32 (14.5%)	0.550
Senior (51–65 years old), *n* (%)	75 (21.6%)	39 (30.7%)	36 (16.4%)	0.149
Education level, *n* (%)				
Low	36 (10.4%)	11 (8.7%)	25 (11.4%)	0.811
Middle	77 (22.2%)	29 (22.8%)	48 (21.8%)	0.919
High	234 (67.4%)	87 (68.5%)	147 (66.8%)	0.789
Job category, *n* (%)				
Agriculture, forestry, fishing	7 (2%)	2 (1.6%)	5 (2.3%)	0.698
Industry	17 (4.9%)	9 (7.1%)	8 (3.6%)	0.758
Production and distribution of electricity, natural gas, steam, and cooled air trade	1 (0.3%)	0 (0.0%)	1 (0.5%)	-
Construction industry	5 (1.4%)	2 (1.6%)	3 (1.4%)	0.987
Wholesale and retail trade, repair of cars	17 (4.9%)	11 (8.7%)	6 (2.7%)	0.644
Transportation and storage	16 (4.6%)	13 (10.2%)	3 (1.4%)	0.634
Hospitality, catering industry	33 (9.5%)	12 (9.4%)	21 (9.5%)	0.993
Information and communication	27 (7.8%)	11 (8.7%)	16 (7.3%)	0.896
Financial services	14 (4.0%)	7 (5.5%)	7 (3.2%)	0.839
Rental and trade of real estate	1 (0.3%)	0 (0.0%)	1 (0.5%)	-
Advice, research, and other specialist business services	19 (5.5%)	4 (3.1%)	15 (6.8%)	0.788
Rental of movable property and other business services	1 (0.3%)	1 (0.8%)	0 (0.0%)	-
Public administration, government, social insurance	15 (4.3%)	5 (3.9%)	10 (4.5%)	0.789
Education	36 (10.4%)	14 (11.0%)	22 (10.0%)	0.925
Health and welfare care	80 (23.1%)	17 (13.4%)	63 (28.6%)	0.204
Culture, sports, and recreation	22 (6.3%)	6 (4.7%)	16 (7.3%)	0.831
Households as employees	1 (0.3%)	0 (0.0%)	1 (0.5%)	-
Other services	35 (10.1%)	13 (10.2%)	22 (10.0%)	0.985
Work characteristics, mean (SD)				
Hours worked per week	29.0 (14.3)	32.8 (15.9)	26.8 (12.7)	<0.001 *
Days worked per week	4.2 (1.9)	4.6 (2.3)	4.0 (1.6)	0.028 *
Days worked on location per week	3.7 (1.6)	3.9 (1.8)	3.5 (1.5)	0.020 *
Days worked from home per week	0.6 (1.4)	0.7 (1.6)	0.5 (1.3)	0.889

Significant sex differences (*p* < 0.05) are indicated by *. ‘-’ indicates that a *p*-value cannot be computed, since one of the cells is empty.

**Table 2:** Absenteeism, presenteeism, and work performance.

Variables Assessed	Overall	Men	Women	*p*-Value
Hangover severity on absenteeism days	6.6 (3.2)	6.4 (3.2)	6.8 (3.2)	0.729
Hangover severity on presenteeism days	4.3 (1.9)	4.0 (1.8)	4.5 (2.0)	0.130
Number of absenteeism days	0.2 (0.9)	0.4 (1.5)	0.1 (0.4)	0.215
Number of presenteeism days	8.3 (39.1)	7.6 (34.4)	8.7 (41.6)	0.323
Performance level when hungover (%)	75.1 (35.3)	73.4 (36.5)	76.1 (34.6)	0.599

**Table 3:** Absenteeism, presenteeism, and work performance according to career level.

Career Level	Junior	Middle	Senior
Age range (years)	18–34	35–50	51–65
*n*	214	58	78
Hangover severity on absenteeism day	6.8 (3.1)	3.8 (4.3)	7.4 (2.4)
Hangover severity on presenteeism day	4.3 (1.9)	3.8 (2.1)	5.0 (2.5)
Number of absenteeism days	0.3 (1.1)	0.1 (0.7) *	0.1 (0.5)
Number of presenteeism days	4.3 (19.5)	11.9 (50.3) *	16.9 (62.9) *
Performance level (%) when hungover	72.8 (33.3)	80.7 (37.6) *	80.0 (37.9) *

Significant differences (*p* < 0.025, after Bonferroni’s correction) from the junior career level are indicated by *.

**Table 4:** Absenteeism, presenteeism, and work performance according to education level.

Education Level	Low	Middle	High
*n*	36	77	234
Hangover severity on absenteeism days	5.3 (3.8)	4.3 (3.4)	7.2 (2.9)
Hangover severity on presenteeism days	4.7 (3.5)	3.6 (1.8)	4.3 (1.9)
Number of absenteeism days	0.03 (0.2)	0.05 (0.3)	0.3 (1.1)
Number of presenteeism days	27.6 (78.2)	2.0 (7.0)	7.4 (35.5)
Performance level (%) when hungover	68.1 (46.1)	79.6 (34.4)	74.7 (33.6)

No significant differences (*p* < 0.025, after Bonferroni’s correction for multiple comparisons) compared to the low education level were found.

## Data Availability

The survey and data are available upon request from the corresponding author.
